# Unhealthy food consumption and its determinants among children aged 6–23 months in Bangladesh: insights from the Demographic and Health Survey 2022

**DOI:** 10.1186/s12889-025-23668-2

**Published:** 2025-07-18

**Authors:** Rafid Hassan, Md. Shahadoth Hossain, Md. Jarif Mahbub, Md. Ruhul Amin, Sanjib Saha

**Affiliations:** 1 Nutrition Research Division, International Centre for Diarrhoeal Disease Research, Bangladesh (icddr,b), Dhaka, Bangladesh; 2 Institute of Nutrition and Food Science, University of Dhaka, Dhaka, 1000 Bangladesh; 3 National Academy for Planning and Development (NAPD), Ministry of Public Administration, Dhaka, 1205 Bangladesh; 4 Department of Clinical Science (Malmo), Lund University, Lund, Sweden

**Keywords:** Unhealthy food consumption, Complementary feeding, Children, BDHS, Bangladesh

## Abstract

**Background:**

Low- and middle-income countries (LMICs), including Bangladesh, are experiencing a nutritional transition characterized by rising unhealthy food consumption (UFC), which increases the risk of nutrient deficiencies and chronic diseases in children. Despite this concern, research on UFC among Bangladeshi children aged 6–23 months is limited. Therefore, this study aims to estimate the prevalence and identify the factors contributing to UFC in this age group.

**Methods:**

This study analyzed the Bangladesh Demographic and Health Survey (BDHS) 2022 dataset, including 2,499 children aged 6–23 months. UFC was defined as the consumption of sweetened beverages or sentinel unhealthy foods within the past 24 hours. Multivariate logistic regression was used to identify factors associated with UFC.

**Results:**

The prevalence of UFC among children was 61.8%, with 49.2% consuming sentinel unhealthy foods and 31.4% consuming sweetened beverages. The strongest predictor of UFC was older child aged 18–23 months (AOR: 3.31, 95% CI: 2.55–4.32), and consuming minimum diversified diet (AOR: 2.50, 95% CI: 1.98–3.15). Other significant factors included recent morbidity (AOR: 1.24, 95% CI: 1.01–1.53), maternal employment (AOR: 1.36, 95% CI: 1.04–1.77), media exposure (AOR: 1.28, 95% CI: 1.02–1.59), and lower paternal education [primary (AOR: 1.72, 95% CI: 1.21–2.44); secondary (AOR: 1.58, 95% CI: 1.14–2.18)]. However, maternal decision-making power (AOR: 0.75, 95% CI: 0.58–0.96) and intended pregnancies (AOR: 0.76, 95% CI: 0.59–0.97) were associated with lower odds of UFC. Regional disparities were observed, with higher UFC prevalence in Dhaka, Khulna, Mymensingh, Rajshahi, and Rangpur.

**Conclusion:**

The study highlights Bangladeshi children’s high prevalence of UFC, which demands public health interventions together with integrating behavior change communication into health, community and family-level services.

## Introduction

Low-and middle-income countries (LMICs) are experiencing a nutritional transition characterized by a shift in dietary patterns toward increased consumption of unhealthy foods, including sugar-sweetened beverages, unhealthy fats, and refined carbohydrates [1]. This transition has been fueled by the global availability of processed foods and has led to the growing prevalence of unhealthy snack foods and beverages in the diets of young children [2, 3]. Such dietary changes contribute to the triple burden of malnutrition in children, encompassing undernutrition, deficiency of micronutrients, and risk of overweight or obesity [4]. Unhealthy food and beverage consumption is linked to poor diet quality and a higher likelihood of developing long-term health issues, such as non-communicable diseases (NCDs) [5–8].

Complementary feeding, typically spanning 6–23 months, is a critical period in a child’s life when breast milk is supplemented with nutrient-dense foods to meet growing nutritional demands [9]. A diverse and healthy diet during this phase is essential for optimal growth, brain development, and immunity [10]. Additionally, this phase also represents a window of opportunity to establish lifelong eating habits and prevent malnutrition [11, 12]. However, the introduction of unhealthy foods during complementary feeding often displaces nutrient-rich options, increasing the risk of malnutrition [11, 13]. In LMICs, the quality of complementary feeding is often compromised by poor dietary diversity and the inclusion of commercial snack foods [14]. These practices contribute to widespread micronutrient deficiencies and the growing double burden of malnutrition, particularly in South Asia, which is home to the highest number of undernourished children (280.9 million) and individuals with micronutrient deficiencies (99 million) [15, 16].

Bangladesh, a South Asian country, has a longstanding history of contending with child undernutrition and insufficient complementary feeding practices. Only 39% of children aged 6–23 months consumed a sufficiently diverse diet, and a mere 29% meet the minimum acceptable diet (MAD) standards [17]. The percentage of children meeting MAD criteria has decreased significantly, dropping from 35% in 2017–18 to 29% in 2022 [17]. Furthermore, the recent Bangladesh Demographic and Health Survey (BDHS) highlighted concerning dietary habits, showing that 32% of children aged 6–23 months consumed sweet beverages, while 49% ate unhealthy sentinel foods the day prior to the survey [17]. These poor dietary patterns contributed to a high prevalence of stunting (21%), underweight (18%), and wasting (10%) in children under two years of age [17]. It also contributed to the widespread micronutrient deficiencies among children. Over half of Bangladeshi children suffered from at least one core micronutrient deficiency, such as vitamin A, zinc, or iron [16, 18]. Despite this alarming situation, research on unhealthy food consumption (UFC) among young children aged 6 to 23 months in Bangladesh remains scarce. Existing studies have predominantly focused on rural settings and have not explored national-level patterns [19, 20]. This highlights a critical need for updated, nationally representative evidence to understand the prevalence and determinants of UFC in this population.

UFC in children’s diets has been identified as a critical indicator for assessing diet quality. Recent updates to the infant and young child feeding (IYCF) guidelines have introduced two new indicators: consumption of sentinel foods and sweet beverages [13]. These foods are linked to higher energy intake and lower micronutrient consumption. Children often exhibit a preference for sugary and salty foods. The introduction of these items during early developmental stages can lead to increased consumption of unhealthy options while discouraging healthier choices [21]. This dietary pattern can contribute to poor nutrition and associated health issues among children. Several factors have been identified as influencing UFC among children in LMICs. These include child-related factors such as age [19, 22] and gender [19, 23]. Parental characteristics, including parental education [19, 20, 23–27], occupation [24], maternal dietary habit [20], antenatal care visits [19, 24, 28], postnatal checkups [27], and media exposure [27] also play a significant role. Additionally, factors such as household wealth or income [5, 20, 23, 27], family size [26], and urban residence [26] further contribute to these dietary patterns. Moreover, the higher cost of healthier foods compared to unhealthy alternatives exacerbates the issue, limiting access to nutritious diets for many families [15].

Therefore, this study aims to estimate the prevalence and factors of UFC among Bangladeshi children aged 6–23 months, using a nationally representative data from BDHS 2022. Findings from this study are expected to guide policymakers, program designers, and health practitioners in promoting healthier child feeding practices and nutrition-sensitive interventions in Bangladesh and similar settings.

## Methods

### Data source

**Fig. 1:**
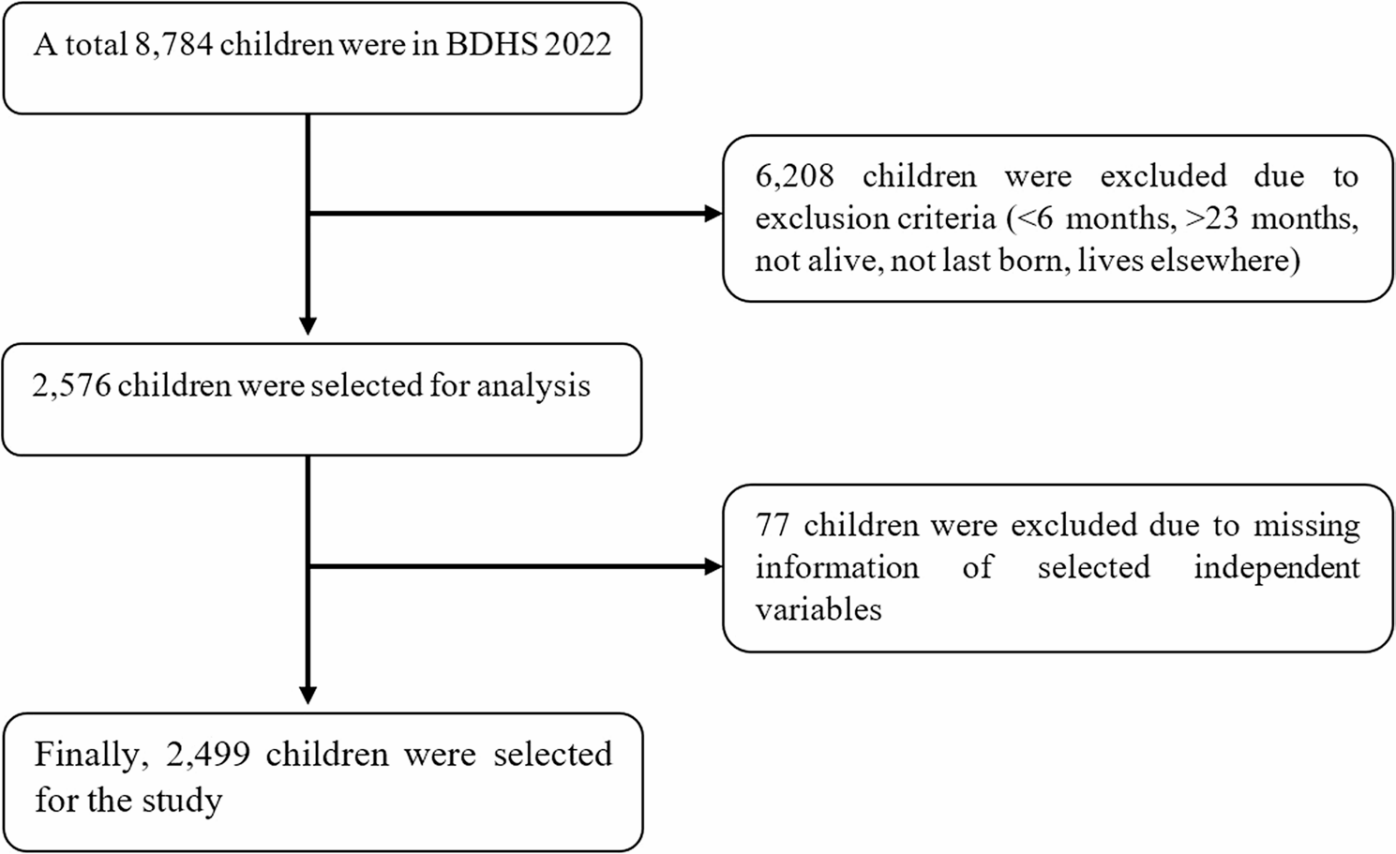
Schematic diagram of sample selection

This study used secondary data from the BDHS-2022, a nationally representative cross-sectional survey. The survey used a two-stage stratified sampling method to ensure representation of the entire population living in non-institutional households throughout Bangladesh. The initial phase involved selecting 675 enumeration units (clusters) through a probability proportional to size sampling method, followed by a complete household listing within each selected unit. In the second stage, 30 households were systematically sampled from each enumeration unit. The survey ultimately gathered data from 8,784 children across 675 clusters nationwide. However, the final sample included 2,499 children aged 6–23 months (Fig. 1). Details of the sampling procedure can be found elsewhere [17].

### Outcome variable

The outcome variable in this study was the UFC, defined as whether children had consumed sweet beverages or selected sentinel unhealthy foods during the day or night prior to the survey [26]. Sweet beverages included items such as juice, tea or coffee/herbal drinks, tinned, powdered, or fresh milk, yogurt, soy milk, chocolate-flavored drinks, sodas, malt, sports, or energy drinks, and other sweetened liquids. Sentinel unhealthy foods encompassed chocolates, sweets, candies, pastries, chips, crisps, French fries, fried dough, instant noodles, and similar items [13].

### Explanatory variables

**Fig. 2:**
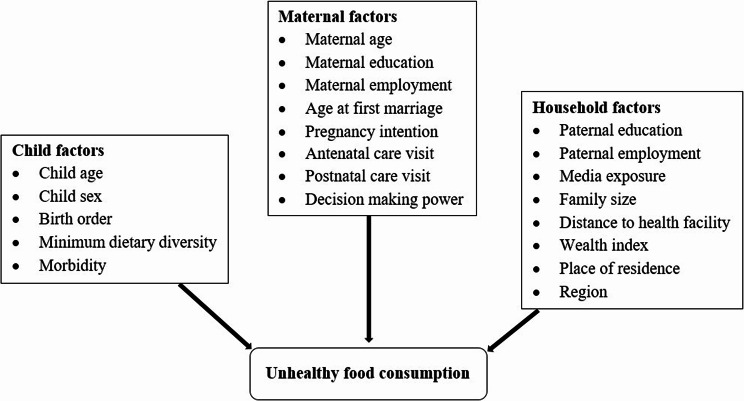
Conceptual framework of factors associated with UFC among children

A conceptual framework has been developed to identify factors at the child, maternal and household levels that may influence UFC in children, based on earlier studies and their availability in BDHS dataset [17, 20, 22–26, 29] (Fig. 2).

Child related characteristics including child age in months (6–11, 12–17, 18–23), sex, birth order (first, second, third or more), recent morbidity, minimum dietary diversity; maternal characteristics including maternal age in years (15–19, 20–24, 25–29, ≥ 30), education (no education, primary, secondary, higher), current employment status (unemployed, employed), age at first marriage in years (< 19, 20–24, ≥ 25), wanted pregnancy (no, yes), antenatal care (ANC) visit (< 4 times, ≥ 4 times), postnatal care (PNC) visit (no, yes), decision making power (low, medium, high); household related factors including paternal education (no education, primary, secondary, higher), paternal employment (unemployed, agricultural, non-agricultural), access to media (no, yes), family size (< 5 members, ≥ 5 members), distance to health facility (big problem, not a big problem), wealth index (poorest, poorer, middle, richer, richest), place of residence (urban, rural), region (Barisal, Chattogram, Dhaka, Khulna, Mymensingh, Rajshahi, Rangpur, Sylhet). Minimum dietary diversity was determined by evaluating whether the child consumed foods from a minimum of five out of eight specific food groups (breastfeeding, grains/roots/tubers, legumes/nuts, dairy products, flesh foods, eggs, fruits and vegetables high in vitamin A, and other fruits and vegetables) during the day prior to the survey [13]. Women’s decision-making power was assessed using the SWPER Global Index, a validated tool for measuring women’s empowerment across three domains: attitude toward violence, social independence, and decision-making [30]. Specifically, the decision-making domain evaluates women’s ability to make decisions regarding health care, major household purchases, and visits to family or relatives. Based on standardized cut-off values, this domain is categorized into three levels: low, medium, and high. Further details on the justification and calculation can be found elsewhere [30]. Recent morbidity was defined as a child experiencing any of the following illnesses—diarrhea, fever, or cough—within the two weeks preceding the survey. Media access was measured based on exposure to any of the following media—newspapers, radio, or television—at least once a week [17].

### Statistical analysis

Descriptive analyses were performed to summarize the data, utilizing frequencies and percentages. To evaluate differences in the prevalence of UFC across various sociodemographic characteristics, the chi-square test of independence was employed. Logistic regression analysis was performed to determine factors associated with UFC, constructing four distinct models: (a) child-related factors, (b) maternal-related factors, (c) household-related factors, and (d) a full model that combined child, maternal, and household-related factors. Adjusted odds ratios (AORs) with 95% confidence intervals (CIs) were reported for each of the model. The variance explained by each model was assessed using R² values. The fitness of each of the regression model was assessed using the Hosmer-Lemeshow test for goodness-of-fit. Multicollinearity among covariates was assessed using the variance inflation factor (VIF), with a threshold of VIF < 10 [31]. All analyses accounted for clustering, stratification, and sampling weights to reflect the complex survey design. Cases with incomplete data were excluded from the analysis. A *p*-value < 0.05 was considered statistically significant. Data analysis was conducted using STATA version 15.

## Results

### Background characteristics

The study included 2,499 children aged 6–23 months, with 38.2% aged 6–11 months. Only one-third of children met the minimum dietary diversity criteria. Among maternal characteristics, 15% were adolescents (15–19 years), 5% had no formal education, 23% had primary education, and 77.5% were unemployed. Approximately 80% of pregnancies were reported as wanted, and half of mothers had no media access. Access to healthcare services was limited—around 41% attended four or more ANC visits, and received PNC. Additionally, around one-fifth of the women had low decision-making power. Most participants (74%) resided in rural areas (Table 1).

**Table 1:** Background characteristics of study participants and associated prevalence of UFC among children

Characteristics	% (n)	Prevalence (% [95%CI])	*P* value^a^
Child related factors			
Child age in months			
6–11	38.2 (954)	47.2 [43.4, 51.1]	<0.001
12–17	28.6 (715)	64.7 [60.9, 68.3]	
18–23	33.2 (830)	76.3 [72.5, 79.7]	
Sex of the children			
Male	50.2 (1266)	62.2 [58.9, 65.4]	0.722
Female	49.8 (1233)	61.4 [58.2, 64.5]	
Birth order			
First	38.7 (936)	62.1 [58.4, 65.8]	0.711
Second	33.4 (862)	62.6 [58.9, 66.1]	
Third or more	27.9 (701)	60.5 [56.5, 64.3]	
Recent morbidity			
No	50.8 (1260)	60.4 [57.0, 63.6]	0.227
Yes	49.2 (1239)	63.3 [59.9, 66.5]	
Minimum dietary diversity			
No	62.8 (1556)	53.0 [50.1, 55.9]	<0.001
Yes	37.2 (943)	76.6 [73.1, 79.8]	
Maternal related factors			
Maternal age in years			
15-19	15.2 (361)	64.0 [57.6, 69.8]	0.387
20-24	33.8 (816)	60.3 [56.5, 63.9]	
25-29	25.9 (688)	64.1 [60.1, 68.0]	
≥30	25.1 (634)	60.2 [55.8, 64.4]	
Maternal education			
No education	5.0 (125)	58.7 [48.8, 68.0]	0.555
Primary	23.0 (575)	60.0 [55.7, 64.3]	
Secondary	52.7 (1318)	61.9 [58.4, 65.3]	
Higher	19.3 (481)	64.6 [59.6, 69.3]	
Maternal employment			
Unemployed	77.5 (1971)	59.8 [57.1, 62.5]	0.002
Employed	22.5 (528)	68.6 [64.0, 72.8]	
Age at first marriage			
<19 years	82.1 (2051)	62.1 [59.5, 64.5]	0.549
20-24 years	13.6 (340)	61.8 [56.0, 67.3]	
25-49 years	4.3 (108)	55.8 [44.4, 66.7]	
Wanted pregnancy			
No	19.7 (493)	66.6 [61.9, 70.9]	0.023
Yes	80.3 (2006)	60.7 [58.1, 63.2]	
ANC visit			
<4 times	59.4 (1459)	61.3 [58.3, 64.1]	0.557
≥4 times	40.6 (1040)	62.6 [59.0, 66.1]	
PNC visit			
No	58.7 (1442)	63.0 [60.1, 65.8]	0.223
Yes	41.3 (1048)	60.1 [56.1, 63.9]	
Decision making power			
Low	18.7 (489)	62.5 [57.8, 67.1]	<0.001
Medium	25.5 (637)	69.3 [65.1, 73.3]	
High	55.8 (1373)	58.1 [55.1, 61.1]	
Household related factors			
Paternal education			
No education	15.3 (378)	54.4 [48.9, 59.8]	0.017
Primary	30.0 (746)	64.2 [60.2, 68.1]	
Secondary	34.7 (849)	64.1 [60.0, 67.9]	
Higher	20.0 (526)	60.0 [55.0, 64.8]	
Paternal employment			
Unemployed	2.3 (58)	54.2 [39.2, 68.5]	0.605
Agricultural	19.1 (472)	62.1 [56.6, 67.3]	
Non-agricultural	78.6 (1969)	62.0 [59.3, 64.5]	
Access to media			
No	53.7 (1336)	58.7 [55.4, 61.8]	0.004
Yes	46.3 (1163)	65.4 [62.1, 68.7]	
Family Size			
<5 members	32.8 (778)	62.8 [58.7, 66.7]	0.526
≥5 members	67.2 (1721)	61.3 [58.6, 64.0]	
Distance to health facility			
Big problem	45.0 (1113)	58.5 [55.0, 62.0]	0.012
Not a big problem	55.0 (1386)	64.5 [61.4, 67.5]	
Wealth index			
Poorest	21.0 (525)	62.0 [57.0, 66.8]	0.341
Poorer	20.8 (520)	57.7 [52.9, 62.3]	
Middle	19.5 (488)	61.4 [56.6, 66.0]	
Richer	19.5 (487)	63.6 [57.2, 69.5]	
Richest	19.2 (479)	65.0 [59.8, 70.0]	
Place of residence			
Urban	26.4 (808)	64.8 [60.1, 69.3]	0.139
Rural	73.6 (1691)	60.7 [58.0, 63.4]	
Division			
Barisal	6.0 (264)	48.1 [41.5, 54.7]	<0.001
Chattogram	21.5 (414)	51.7 [46.3, 57.1]	
Dhaka	24.0 (363)	63.7 [58.3, 68.7]	
Khulna	10.3 (280)	72.5 [65.8, 78.4]	
Mymensingh	9.2 (323)	65.0 [59.1, 70.5]	
Rajshahi	10.3 (247)	65.6 [58.1, 72.4]	
Rangpur	11.9 (306)	73.1 [66.3, 79.0]	
Sylhet	6.8 (302)	53.0 [47.4, 58.5]	

^a^*P* values were derived from chi-square test of independence where row percentage was employed

### Prevalence of UFC

**Fig. 3:**
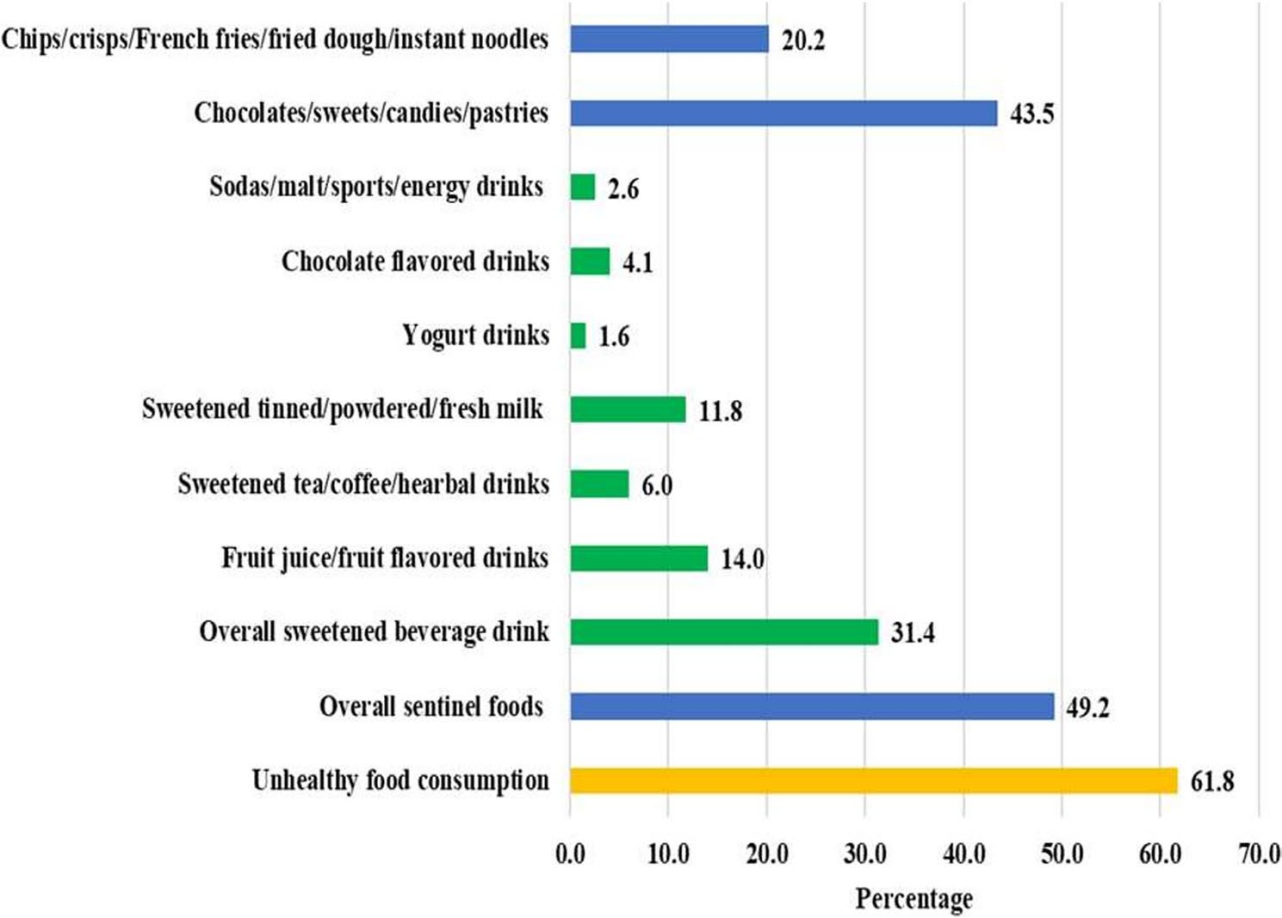
Prevalence of UFC among children aged 6 to 23 months

Overall, the prevalence of UFC among children was 61.8%. Nearly half (49.2%) of the children consumed sentinel foods, while 31.4% consumed sweetened beverages. Among specific types of sweetened drinks, 14.0% consumed fruit juice or fruit-flavored drinks, 11.8% consumed sweetened milk, and 6.0% consumed sweetened tea, coffee, or herbal drinks. Consumption of other liquid options, such as yogurt drinks (1.6%), chocolate-flavored drinks (4.1%), and sodas, malt, sports, or energy drinks (2.6%), was relatively low. Consumption of sweetened solid foods was also prevalent, with 43.5% of children consuming chocolates, sweets, candies, or pastries. Additionally, 20.2% consumed fried or processed snacks such as chips, crisps, French fries, fried dough, or instant noodles (Fig. 3).

The prevalence of UFC varied significantly across several background characteristics (Table 1). Child age was associated with UFC, with prevalence increasing significantly from 47.2% among children aged 6–11 months to 76.3% among those aged 18–23 months (*p* < 0.001). Children who met the minimum dietary diversity criteria had a significantly higher tendency to eat unhealthy foods (76.6%) compared to those who did not (53.0%, *p* < 0.001). Children of employed mothers had higher UFC (68.6%) compared to those of unemployed mothers (59.8%, *p* = 0.002). Additionally, children from unwanted pregnancies showed a higher prevalence of UFC (66.6%) compared to those from wanted pregnancies (60.7%, *p* = 0.026). Children of mothers with high decision-making power had a significantly lower percentage of UFC (*p* < 0.001). The prevalence of UFC was lowest among children of fathers with no education (54.4%, *p* = 0.017). Media access was associated with an increased UFC (65.4% vs. 58.7%, *p* = 0.004). Prevalence of UFC was highest among children of mothers who reported distance to the health facility as not a big problem (64.5%, *p* = 0.012). Geographical variation was evident, with the prevalence of UFC differing significantly by division (*p* < 0.001). The highest prevalence was observed in Rangpur (73.1%) and Khulna (72.5%), while the lowest was in Barisal (48.1%) and Sylhet (53.0%).

### Factors associated with UFC among children

Table 2 presents the associated factors of UFC among children aged 6 to 23 months. Children aged 12–17 months (AOR: 1.86, 95% CI: 1.48–2.35, *p* < 0.001) and 18–23 months (AOR: 3.31, 95% CI: 2.55–4.32, *p* < 0.001) had significantly higher odds of consuming unhealthy foods compared to those aged 6–11 months. Children with recent morbidity had significantly higher odds of consuming UFC compared to those without morbidity (AOR = 1.24, 95% CI: 1.01–1.53; *p* = 0.044). Consumption of a minimum diversified diet was also associated with increased odds of UFC (AOR: 2.50, 95% CI: 1.98–3.15, *p* < 0.001). Maternal employment was also associated with higher odds of UFC (AOR: 1.36, 95% CI: 1.04–1.77, *p* = 0.023). Pregnancy intention was significantly associated with UFC; children of mothers who wanted the pregnancy had 24% lower odds of consuming unhealthy foods (AOR: 0.76, 95% CI: 0.59–0.97, *p* = 0.031) compared to those whose pregnancies were unplanned. High maternal decision-making power was associated with 25% lower odds of UFC (AOR: 0.75, 95% CI: 0.58–0.96, *p* = 0.022). Children with fathers who had primary (AOR: 1.72, 95% CI: 1.21–2.44, *p* = 0.003) or secondary (AOR: 1.58, 95% CI: 1.14–2.18, *p* = 0.006) education were more likely to consume unhealthy foods than those with fathers who had higher education. Media access was associated with 28% higher odds of consuming unhealthy foods among children (AOR: 1.28, 95% CI: 1.02–1.59, *p* = 0.031). Regional disparities were evident, with children in Dhaka (AOR: 1.51, 95% CI: 1.03–2.22, *p* = 0.037), Khulna (AOR: 2.27, 95% CI: 1.47–3.51, *p* < 0.001), Mymensingh (AOR: 1.73, 95% CI: 1.14–2.63, *p* = 0.010), Rajshahi (AOR: 1.81, 95% CI: 1.13–2.91, *p* = 0.014), and Rangpur (AOR: 2.78, 95% CI: 1.77–4.35, *p* < 0.001) having significantly higher odds of UFC compared to children in Barisal.

**Table 2:** Factors associated with UFC among children aged 6–23 months

Characteristics	Child factors		Maternal factors		Household factors		Full model	
	AOR [95%CI]	*P*-value	AOR [95%CI]	*P*-value	AOR [95%CI]	*P*-value	AOR [95%CI]	*P*-value
Child age in months								
6–11	Ref.						Ref.	
12–17	1.79 [1.43-2.25]	<0.001					1.86 [1.48-2.35]	<0.001
18–23	3.08 [2.38-4.01]	<0.001					3.31 [2.55-4.32]	<0.001
Sex of the children								
Male	Ref.						Ref.	
Female	1.00 [0.82-1.22]	0.981					1.03 [0.83-1.27]	0.819
Birth order								
First	Ref.						Ref.	
Second	1.09 [0.87-1.35]	0.466					1.12 [0.84-1.49]	0.456
Third or more	0.95 [0.76-1.20]	0.679					1.05 [0.67-1.66]	0.829
Recent morbidity								
No	Ref.						Ref.	
Yes	1.27 [1.03-1.56]	0.025					1.24 [1.01-1.53]	0.044
Minimum dietary diversity								
No	Ref.						Ref.	
Yes	2.52 [2.02-3.15]	<0.001					2.50 [1.98-3.15]	<0.001
Maternal age in years								
15-19			Ref.				Ref.	
20-24			0.87 [0.64-1.19]	0.397			0.74 [0.52-1.05]	0.092
25-29			1.07 [0.78-1.48]	0.676			0.87 [0.55-1.39]	0.567
≥30			0.94 [0.67-1.32]	0.718			0.67 [0.39-1.17]	0.163
Maternal education								
No education			0.71 [0.43-1.17]	0.180			0.92 [0.48-1.74]	0.794
Primary			0.75 [0.56-1.01]	0.058			0.80 [0.54-1.17]	0.244
Secondary			0.82 [0.61-1.10]	0.181			0.79 [0.56-1.12]	0.188
Higher			Ref.				Ref.	
Maternal employment								
Unemployed			Ref.				Ref.	
Employed			1.51 [1.19-1.91]	0.001			1.36 [1.04-1.77]	0.023
Age at first birth								
<19 years			Ref.				Ref.	
20-24 years			0.90 [0.72-1.11]	0.318			1.00 [0.78-1.29]	0.996
25-49 years			0.72 [0.48-1.09]	0.123			0.79 [0.48-1.32]	0.370
Wanted pregnancy								
No			Ref.				Ref.	
Yes			0.79 [0.63-1.00]	0.047			0.76 [0.59-0.97]	0.031
ANC visit								
<4 times			Ref.				Ref.	
≥4 times			1.09 [0.88-1.34]	0.425			0.97 [0.77-1.22]	0.791
PNC visit								
No			Ref.				Ref.	
Yes			0.87 [0.71-1.06]	0.179			0.93 [0.75-1.16]	0.539
Decision making power								
Low			Ref.				Ref.	
Medium			1.30 [0.98-1.72]	0.065			1.24 [0.93-1.65]	0.139
High			0.78 [0.62-0.99]	0.043			0.75 [0.58-0.96]	0.022
Paternal education								
No education					1.00 [0.70-1.43]	0.990	1.29 [0.84-1.97]	0.248
Primary					1.49 [1.10-2.01]	0.010	1.72 [1.21-2.44]	0.003
Secondary					1.39 [1.04-1.85]	0.026	1.58 [1.14-2.18]	0.006
Higher					Ref.		Ref.	
Paternal employment								
Unemployed					Ref.		Ref.	
Agricultural					1.23 [0.65-2.34]	0.518	1.18 [0.63-2.23]	0.600
Non-agricultural					1.25 [0.69-2.26]	0.459	1.20 [0.67-2.13]	0.535
Access to media								
No					Ref.		Ref.	
Yes					1.19 [0.97-1.46]	0.097	1.28 [1.02-1.59]	0.031
Family Size								
<5 members					Ref.		Ref.	
≥5 members					1.00 [0.82-1.22]	0.998	0.96 [0.76-1.21]	0.738
Distance to health facility								
Big problem					Ref.		Ref.	
Not a big problem					1.18 [0.97-1.44]	0.103	1.24 [1.00-1.54]	0.053
Wealth index								
Poorest					Ref.		Ref.	
Poorer					0.79 [0.58-1.07]	0.130	0.74 [0.53-1.03]	0.076
Middle					0.90 [0.65-1.26]	0.553	0.92 [0.63-1.35]	0.669
Richer					0.97 [0.66-1.45]	0.899	0.97 [0.64-1.49]	0.901
Richest					1.08 [0.71-1.64]	0.729	0.96 [0.59-1.55]	0.861
Place of residence								
Urban					Ref.		Ref.	
Rural					0.87 [0.68-1.11]	0.262	0.84 [0.64-1.09]	0.182
Division								
Barisal					Ref.		Ref.	
Chattogram					1.12 [0.79-1.59]	0.514	1.15 [0.78-1.70]	0.491
Dhaka					1.65 [1.16-2.34]	0.005	1.51 [1.03-2.22]	0.037
Khulna					2.62 [1.74-3.97]	<0.001	2.27 [1.47-3.51]	<0.001
Mymensingh					2.04 [1.41-2.95]	<0.001	1.73 [1.14-2.63]	0.010
Rajshahi					1.97 [1.29-3.01]	0.002	1.81 [1.13-2.91]	0.014
Rangpur					2.85 [1.88-4.33]	<0.001	2.78 [1.77-4.35]	<0.001
Sylhet					1.18 [0.84-1.66]	0.344	1.25 [0.85-1.84]	0.263
R^2^	13.7%		3.1%		5.5%		21.2%	

*AOR* Adjusted Odd Ratios, *CI* Confidence Interval, *Ref.* Reference

## Discussion

The present study analyzed the recent BDHS 2022 dataset to estimate the prevalence and determinants associated with UFC among Bangladeshi children aged 6–23 months. The findings revealed that nearly two-thirds of children in this age group consumed unhealthy foods. This study also identified multiple factors associated with UFC, including child characteristics (age, minimum dietary diversity, and recent illness), maternal factors (employment, decision-making power, and pregnancy intention), and household-level factors (paternal education, media exposure, and geographic region).

The high prevalence of UFC observed in this study aligns with earlier studies conducted in rural Bangladesh (62%) [20], Cambodia (55%) [32], Nepal (74%) [2], and African countries (62.4%) [28]. This consistency suggests the widespread prevalence of unhealthy dietary practices in similar demographic contexts. However, a study among rural Bangladeshi children reported a lower prevalence, with only 12.2% consuming unhealthy snacks and 2.5% consuming sweet beverages [19]. This disparity could be attributed to differences in study populations, study periods, geographic settings, socio-economic conditions, definitions, or data collection methods. The earlier study was conducted in a rural area in 2018, had a smaller sample size, and preceded the introduction of the 2021 WHO guidelines for infant and child feeding practices, which were used in the present study. These variations in methodology and contextual factors likely contributed to the observed discrepancies.

In line with earlier literature in Bangladesh and other LMICs, this study revealed that older children had higher odds of UFC compared to younger ones [19, 27]. As children grow older, their dietary habits tend to shift towards consuming more unhealthy foods due to a combination of environmental, behavioral, and socioeconomic factors. Parents and caregivers may introduce more convenient and accessible foods, which often lack nutritional quality, driven by affordability, time constraints, and the perceived palatability of these options among young children [33, 34]. Additionally, early exposure to unhealthy foods can shape children’s taste preferences, making them more inclined toward these foods as they age [35].

This study found that children with recent morbidity were more likely to consume unhealthy foods than those without, aligning with prior research in Bangladesh [36]. This might be the alteration of taste preferences during illness, leading children to favor sweeter or saltier foods, which are typically found in processed snacks and sugary treats [21]. Parents, in their effort to comfort sick children and encourage eating despite a reduced appetite, often resorted to these more appealing but less nutritious options. When children refused other foods, parents frequently offered sweets [36].

Contrary to the expectation that dietary diversity supports healthier eating, this study found that children who consumed a minimum diversified diet had higher odds of UFC. This finding aligned with earlier studies conducted in rural Bangladesh [20, 37]. One possible explanation is that increased dietary diversity reflects greater access to a wider variety of foods—including commercially produced and ultra-processed items. In contexts where household economic conditions are improving and packaged foods are more readily available—due to local production and aggressive food marketing—diversifying a child’s diet may inadvertently include foods high in sugar, salt, and unhealthy fats [38]. As a result, the concept of dietary diversity may not always equate to dietary quality in environments where unhealthy options are prevalent.

Maternal employment was associated with increased consumption of UFC among children. This finding parallels studies in LMICs where maternal employment outside the home has often correlated with greater reliance on commercially available, convenient foods to save time [39, 40]. Working mothers may lack the opportunity or resources to prepare healthier homemade options, thus opting for readily available packaged foods. Moreover, increased work hours can result in children spending more time unsupervised, which may promote unhealthy eating behaviors such as snacking on junk food and engaging in sedentary activities like watching television [41].

Maternal higher decision-making power was significantly associated with lower intake of unhealthy foods among children. Maternal autonomy in decision-making fosters healthier food availability at home, which directly influences children’s eating patterns [42]. Consistent with previous research, maternal empowerment, particularly in decision-making, is linked to better nutritional outcomes for children, likely due to a greater ability to prioritize healthy food options and access information, resources, and support [43, 44].

The role of parental education in influencing UFC among children is essential, particularly regarding paternal education. Children of fathers with low educational attainment were more likely to consume unhealthy foods, a trend supported by studies in Bangladesh and other South Asian countries that highlighted that higher paternal education increases nutrition awareness, leading to healthier food choices and better adherence to complementary feeding practices [25, 45]. Interestingly, this study found no significant association between maternal education and UFC, which could stem from mothers’ limited autonomy in financial decision-making and food acquisition behaviors. In Bangladesh, men are traditionally responsible for household food purchases and often buy unhealthy sweet foods for children [46]. Fathers frequently use sweets as a quick solution to pacify crying children or to distract them during meal preparation, driven by convenience, affordability, and personal gratification [36]. This behavior reflects the social and cultural norms, emphasizing the need to involve fathers in nutrition education to address UFC effectively.

Similar to earlier study, maternal media exposure was associated with higher odds of UFC in this study [27]. Exposure to television and advertisements has been linked to the promotion of unhealthy food consumption in children [47, 48]. In Bangladesh, media often includes advertisements for sugary snacks and processed foods, which may influence mothers to provide these items to their children [49].

This study identified that children of mothers who intended their pregnancy were found to have lower chances of being fed unhealthy foods. Planned pregnancy might lead to greater attentiveness to healthy feeding practices, likely due to enhanced parental investment in child-rearing and nutrition education. Mothers with planned pregnancies are also more likely to seek prenatal care and resources on recommended feeding practices, fostering healthier dietary patterns for their children [50]. However, unplanned pregnancies often bring challenges such as psychological stress and socioeconomic pressures, which can negatively impact maternal healthcare utilization, feeding decisions and lead to a reliance on convenient, unhealthy food options [51, 52]. A weaker emotional bond between mother and child may also play a role when pregnancies are unintended. Supporting this, a study in India found that children from unintended pregnancies were 11.5% less likely to be exclusively breastfed and 17% less likely to receive full immunizations, with higher prevalence of malnourishment in Bangladesh [53], India [54], and Ethiopia [55].

This study revealed significant regional variations in UFC among children, likely driven by differences in the affordability of healthy diets across various regions. UFC tends to be more affordable and convenient, leading to higher consumption rates. For instance, previous research indicated that households in divisions such as Khulna, Rajshahi, Rangpur, and Mymensingh struggled the most with affording recommended diets, while households in Chattogram experience less difficulty [56]. Additionally, Barisal division has the lowest costs associated with a recommended diet, which may contribute to a lower UFC there. However, Dhaka exhibits a higher availability of unhealthy foods compared to other cities, further explaining the regional disparities in UFC.

This research did not find a significant association between the area of residence and UFC among children. In parallel to this study, high consumption of sugary foods in rural areas indicates the increasing availability and affordability of these foods, which have become more accessible due to improved transportation, urbanization, and changing market dynamics [20, 36, 38]. Additionally, cultural shifts have normalized the inclusion of sugary and unhealthy foods in children’s diets across all regions. This is compounded by economic pressures that drive families to prioritize cost-effective food choices, often resulting in a reliance on cheaper, less nutritious options [36]. Furthermore, the influence of marketing strategies promoting unhealthy foods has penetrated both urban and rural markets, making these choices appealing to parents seeking quick solutions for feeding their children. As a result, UFC has become entrenched in complementary feeding practices throughout Bangladesh, transcending geographic boundaries. Interestingly, this study found no significant association between family wealth status and UFC. This may be due to the widespread availability and marketing of processed and unhealthy foods, making them accessible across all economic groups. While wealthier families can afford items like sweets, chocolates, and cookies, affordable unhealthy options are readily available in Bangladesh, leading to frequent consumption among children from poorer families [20, 36].

This study identifies key opportunities to address UFC among young Bangladeshi children. Given that factors like older age and higher dietary diversity are strong predictors of UFC, it is crucial for policies to prioritize early childhood feeding practices. To promote optimal IYCF practices, educational programs should focus on guiding parents to introduce diverse yet healthy complementary foods. The second national plan of action for nutrition already emphasizes the role of social and behaviour change communication (SBCC) [57]. The successful national programs, such as family planning services and the expanded programme on immunization led by the ministry of health and family welfare (MOHFW), can serve as strategic entry points for these initiatives. Integrating SBCC into all healthcare contacts during pregnancy and the first two years of a child—including antenatal, delivery, and postnatal care, as well as immunization visits, growth monitoring, and child health services—will ensure that caregivers receive consistent guidance. Additionally, community-based support networks, such as mother-to-mother groups and peer counselors, should be strengthened to offer personalized support [58]. Economic interventions, including a health surcharge on unhealthy sugary foods and beverages suggested by World Health Organization—similar to the tax on tobacco—could be implemented [59]. This approach could be implemented by MOHFW and Ministry of Finance. Simultaneously, subsidies for nutrient-dense foods like fruits, vegetables, and whole grains could be expanded through existing social safety net programs. Since early exposure to unhealthy food marketing influences children’s dietary habits, stricter regulations on advertising are essential. The Bangladesh telecommunication regulatory commission and the ministry of information and broadcasting should strengthen advertising standards, particularly those targeting children, while supporting public awareness campaigns that promote healthy eating. Finally, encouraging planned pregnancies and strengthening early maternal counseling through family planning services could improve infant feeding practices.

This study’s primary strength lies in its utilization of a large, nationally representative sample, which ensures the reliability and generalizability of the findings on the prevalence and factors influencing UFC among children in the age group of 6 to 23 months in Bangladesh. Despite its strengths, the study has certain limitations. This study has several limitations that warrant consideration. First, its cross-sectional design and reliance on a single 24-hour dietary recall restrict causal inference and may misclassify habitual dietary intake, as transient or atypical consumption patterns could skew results. The absence of portion-size data further limits the ability to quantify actual food consumption, introducing potential biases from recall inaccuracies and social desirability. Additionally, unmeasured confounders-such as household food security, caregiving responsibilities, or regional food availability-may influence feeding practices but were not accounted for. The operationalization of media exposure, sentinel-food categorization, and maternal decision-making relied on simplified metrics, which may inadequately capture the complexity of feeding behaviors or contextual variability across diverse socio-cultural settings. Future studies should integrate longitudinal designs, repeated dietary assessments, and mixed method approaches to address these gaps and enhance the validity of findings.

## Conclusions

This study sheds light on the widespread prevalence and key determinants of UFC among Bangladeshi children aged 6–23 months. With nearly two-thirds of children in this age group consuming unhealthy foods, the findings emphasize the urgent need to address this public health concern. Influential factors span multiple domains, including child age, minimum dietary diversity, recent illness, maternal employment status, decision-making power, intentional pregnancy, paternal education, media exposure, and geographic region. These insights highlight the importance of comprehensive, multifaceted strategies—such as policy reforms (e.g., sugary food surcharges and marketing restrictions), targeted subsidies for nutritious foods, and SBCC integrated across health, community, and family-level services—to promote healthier dietary practices and long-term child well-being.

## Data Availability

All data are publicly accessible from the DHS database: https://www.dhsprogram.com/Countries/Country-Main.cfm?ctry_id=1&c=Bangladesh&Country=Bangladesh&cn=&r=4.

## Abbreviations

AOR: Adjusted odds ratio
ANC: Antenatal care
BDHS: Bangladesh Demographic and Health Survey
CI: Confidence intervals
IYCF: Infant and Young Child Feeding
LMICs: Low- and middle-income countries
MAD: Minimum acceptable diet
MOHFW: Ministry of health and family welfare
NCDs: Non-communicable diseases
PNC: Postnatal care
SBCC: Social and behaviour change communication
SWPER: Survey-based women’s empowerment index
UFC: Unhealthy food consumption
VIF: Variance inflation factor
